# Effects of di-*n*-butyl phthalate on the physiology and ultrastructure of cucumber seedling roots

**DOI:** 10.1007/s11356-014-2580-x

**Published:** 2014-02-28

**Authors:** Ying Zhang, Yue Tao, Guoqiang Sun, Lei Wang

**Affiliations:** School of Resource and Environment, Northeast Agricultural University, Harbin, 150030 People’s Republic of China

**Keywords:** DBP, Antioxidant enzymes, Cucumber, Ultrastructure

## Abstract

Agricultural pollution caused by the use of plastic sheetings has been documented to be a widespread problem in most of the major crop-planting regions of the world. In order to better understand the phytotoxic mechanisms induced by phthalic acid esters involved with this problem, *Cucumber sativus* L. cv Jinyan No. 4 were sown in pots to the three-leaf-stage in the presence of di-*n*-butyl phthalate (DBP; 0, 30, 50, 100, and 200 mg L^−1^) for 1, 3, 5, or 7 days. Physiology, biochemistry, and ultrastructure of seedling roots were examined. The results indicated that activities of three antioxidant enzymes (superoxide dismutase (SOD), catalase (CAT), and peroxidase (POD)) were stimulated at low-DBP treatments and decreased under higher levels (>100 mg L^−1^) compared to the controls. On the other hand, SOD and POD provided a better defense against DBP-induced oxidative damage in the roots of cucumber seeding, compared to CAT. The productions of both malondialdehyde (MDA) and proline (Pro) were promoted under DBP stress. Visible impact on the cytoderm, mitochondrion, and vacuole was detected, possibly as a consequence of free radical generation. These results suggested that activation of the antioxidant system by DBP led to the formation of reactive oxygen species that resulted in cellular damage.

## Introduction

Phthalic acid esters (PAEs) are mainly used in the plastics industry as adhesives and plasticizers for polyvinyl chloride materials to improve flexibility and easy manufacturing. They are a kind of toxic persistent organic pollutants (Clausen et al. 2004; Gomez-Hens and Aguilar-Caballos 2003a, b; Wensing et al. 2005). They have been found in many environmental samples, such as water, soil (Liang et al. 2010), sediments (Waters et al. 2007), and atmosphere (Zeng et al. 2009; Wang et al. 2012), and even in tissues of higher animals (Russo et al. 2012). Release of PAEs into the environment during manufacture, use, and disposal has caused increasing attention, because some PAEs, as well as their metabolites and degradation products, have been reported to cause adverse effect on health, such as endocrine disruption, hypospadias, reproductive toxicity, malformations, and fatality (Gomez-Hens and Aguilar-Caballos 2003a, b; Inoue et al. 2005; Koch et al. 2005; Sathyanarayana et al. 2008).

In China, there is an extensive use and continuous exploitation of plastic sheeting as agricultural films in most farmlands, in order to yield more farm produce (Wang et al. 2012). Consequently, large amounts of PAE compounds from the plastic films caused a serious threat to agricultural environment (Fromme et al. 2004; Wang et al. 2013; Xu et al. 2008; Kong et al. 2012). di-*n*-butyl phthalate (DBP), one of the important members in the family of PAEs was listed as environmental priority pollutants by the US Environmental Protection Agency (1991), the European Union (1993), and China National Environmental Monitoring Center (Wang et al. 1995). DBP pollutants can enter soils via irrigation and the application of pesticides and plastic film (Michael et al. 1984). As a result, the decrease in the quality of vegetables caused by DBP pollution has posed a potential risk for human health through the food chain. Previous studies have shown that many pollutants are redox-active (Kang et al. 2007; Kreiner et al. 2002). The redox products could attack the cell membrane bringing about oxidative stress to the body and even causing cell death or canceration (Zhang et al. 2003). However, organisms were not attacked in a passive state during the process of the reaction of lipid peroxidation owing to active oxygen free radicals; there was a well-established antioxidant system in their body. For instance, SOD, CAT, and POD are three of the most important antioxidant enzymes, Pro and MDA are two of the antioxidating non-enzyme substances. Enzymatic and non-enzymatic reactions play an important role in removing toxic substances which are generated by chemical mutagen, promote cancer agent, and DNA hydrogen peroxide in normal cells.

Recently, many studies on PAES oxidative effects were mainly focused on various animal and plants cells. However, the biological effects of PAEs on early growth stages of vegetable crops have not been widely investigated until now and only few studies were conducted about the changes in physiology and ultrastructure of plants under PAE stress. Plant roots as a media of absorption and transportation of nutrients have an important effect to plant growth. To investigate the effects of PAEs on plant roots has a profound significance. Cucumber as one of the public favorite vegetables has been selected as a model plant in many studies (Huang et al. 2009). In China, cucumber is usually cultivated in greenhouse, and in a continuous cropping system. Due to the extensive use of plastic films as mulching materials, cucumber was seriously affected by the pollution of PAEs. In this paper, the changes of antioxidant defense system and cell ultrastructure of cucumber seedling roots under DBP stress were investigated. The toxic effect of DBP on the roots of cucumber seedling was analyzed.

## Materials and methods

### Plant materials and stress treatment

Seeds of *Cucumber sativus* L. cv Jinyan No. 4 were planted into planting pots in September at Northeast Agricultural University horticultural garden. The average temperature in the greenhouse was 27 ± 1 °C during the day and was 20 ± 1 °C during the night. The relative humidity was kept at 60–70 %. For DBP treatment and cucumber plants were applied according to the method of Zhang et al. (2014).

### Activities of antioxidant enzymes

Root tissues (0.5 g) were ground to fine powder using a chilled pestle and mortar. Five milliliters of 50 mM potassium phosphate buffer (pH 7.0), 0.2 mM ethylenediamine tetraacetic acid, 1 mM ascorbic acid, and 2 % (*w*/*v*) polyvinylpyrrolidone were added to the powder. The homogenate was centrifuged at 12,000×*g* for 20 min at 4 °C and the supernatant was collected for assays of the activity of enzymes.

The CAT (EC 1.11.1.6) activity was determined by measuring the rate of H_2_O_2_ consumption at 240 nm (Aebi 1984). The reaction mixture contained 50 mM potassium phosphate buffer (pH 7.0), 10.5 mM H_2_O_2_, and 0.2 ml enzyme fraction.

The POD (EC 1.11.1.7) activity was measured based on the method of Pinhero et al. (1997). The reaction mixture contained 50 mM phosphate buffer (pH 7.8), 200 mM H_2_O_2_, 25 mM guaiacol, and 0.2 ml of the supernatant. The molar extinction coefficient of 26.6 mM^−1^ cm^−1^ was used for the enzyme activity calculation. Enzyme activity was defined by the absorbance at 470 nm changes per minute.

The SOD (EC 1.15.1.1) activity was determined by the method of Giannopolitis and Reis (1977). The reaction mixture was prepared by mixing 50 mM phosphate buffer (pH 7.8), 77.12 μM nitro blue tetrazolium, 0.1 M EDTA, 13.37 mM methionine, 0.1 ml enzyme fraction, and 20 μM riboflavin. The mixture was illuminated for 10 min in an aluminum foil-lined box, containing two 20 W fluorescent tubes. A control tube in which the sample was unirradiated was run in parallel. The solution absorbance at 560 nm was recorded using a spectrophotometer. Fifty percent inhibition rate of nitro blue tetrazolium was taken as equivalent to 1 unit of SOD activity.

### Antioxidation non-enzyme substrate assay

The content of MDA was measured according to the method of Chen et al. (2013). About 0.5 g fresh root samples were submerged in 5.0 ml of 5 % (*w*/*v*) trichloroacetic acid in boiling water bath for 10 min and then centrifuged at 12,000×*g* for 10 min. The supernatant was measured at the absorbance of 532, 600, and 450 nm.

The Pro content was determined according to the method described by Shevyakova et al. (2009). Root samples (0.5 g) were homogenized in 5 ml of 3 % (*w*/*v*) sulfosalicylic acid and put in boiling water bath for 10 min, then filtered into a clean test tube after cooling. Two milliliters filtrate was taken to mix with 2 ml acetic acid and 2 ml of 2.5 % (*w*/*v*) acidic ninhydrin for another 30 min, in boiling water bath. Five milliliters toluene was added in the mixture, and then the whole composition was shaken for 30 s. The extract was measured at the absorbance of 520 nm.

### Observations of root ultrastructure

Two roots from each treatment were randomly selected. Small root fragments (about 1 mm^2^ in length) were fixed in 2.5 % (*v*/*v*) glutaraldehyde (pH 7.2) at 4 °C overnight. The samples were washed three times by phosphate-buffered saline solution (PBS) (pH 6.8), and immersed in 2 % (*m*/*v*) osmium tetroxide solution for 4 h in a hood. Subsequently, PBS (pH 6.8) and different concentrations of ethanol solutions were used to rinse and dehydrate the sample. Ethanol was replaced by acetone solution twice. Finally, the specimens were embedded in Spurr’s resin, and polymerized at 60 °C. Ultra-thin sections (80 nm) were collected on copper grids (300 meshes) and double stained with 1.0 % (*w*/*v*) uranyl acetate followed by 5.0 % (*w*/*v*) lead citrate. Samples were observed at 90 kV using an H-600IV transmission electron microscope (Hitachi, Tokyo, Japan).

### Statistical analyses

To test the effects of PAE stress on the activity of enzymes, and the antioxidation non-enzyme substrate contents, a completely random experimental design was adopted. All the experimental data were expressed with five replicates. Data were analyzed by one-way analysis of variance. Mean values were compared by Duncan’s new multiple range test at the 5 % level using the SPSS 19.0 software. The data were represented as mean ± SD.

## Result

**Fig. 1:**
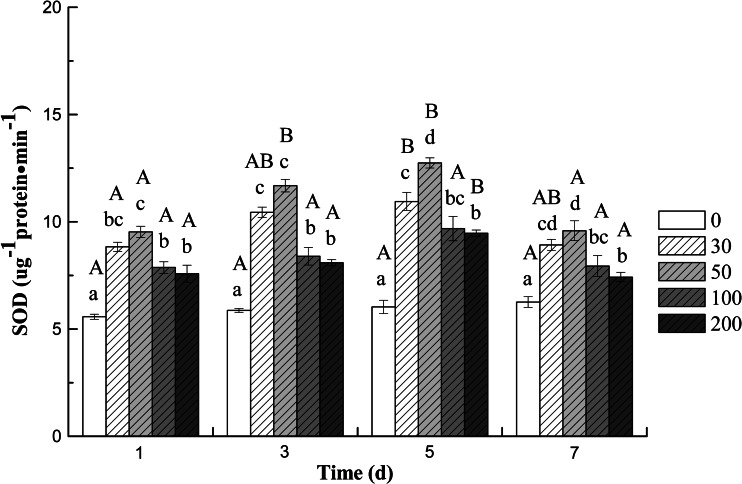
The effect of DBP on the SOD activity of *C. sativus* L. cv Jinyan No. 4

**Fig. 2:**
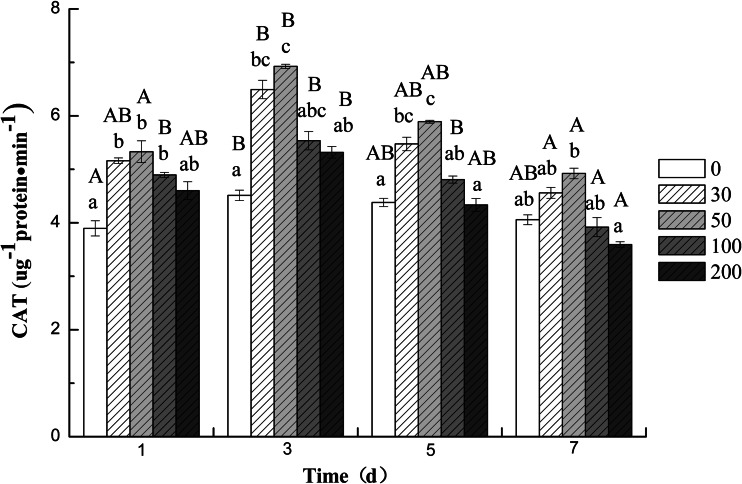
The effect of DBP on the CAT activity of *C. sativus* L. cv Jinyan No. 4

**Fig. 3:**
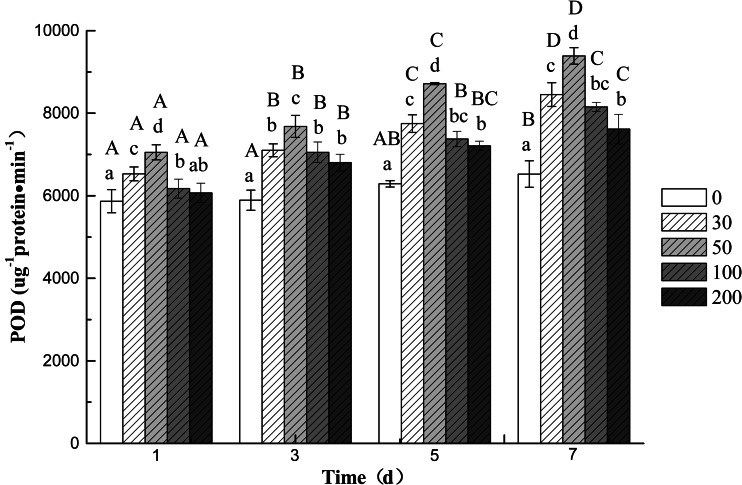
The effect of DBP on the POD activity of *C. sativus* L. cv Jinyan No. 4

The activities of SOD, CAT, and POD in the roots of cucumber seedling varied with different concentrations of DBP and treatment time. The results indicated that compared to the control group, both SOD (Fig. 1) and POD (Fig. 3) activities increased significantly (*p* < 0.05) during whole treatment at all DBP concentrations. The highest activity of SOD and CAT was observed at 5 and 3 days (Fig. 2), respectively, afterwards decrease in activity was observed for the two enzymes after 5 and 7 days, respectively. The results indicated that two antioxidant enzymes’ activities showed an increase at lower DBP concentration and a decline under higher DBP concentration. Application of 50 mg L^−1^ DBP concentration led to a significant increase in activities of antioxidant enzymes (SOD and CAT). The CAT activity was found to be inhibited significantly (*P* < 0.05) when exposed to 100 and 200 mg L^−1^ DBP at 7 days; but inhibition was more severe at 200 mg L^−1^ DBP application. In contrast, the activities of POD increased progressively during the treatment period (1–7 days) compared to that in the roots of plants not exposed to DBP (Fig. 3).

**Fig. 4:**
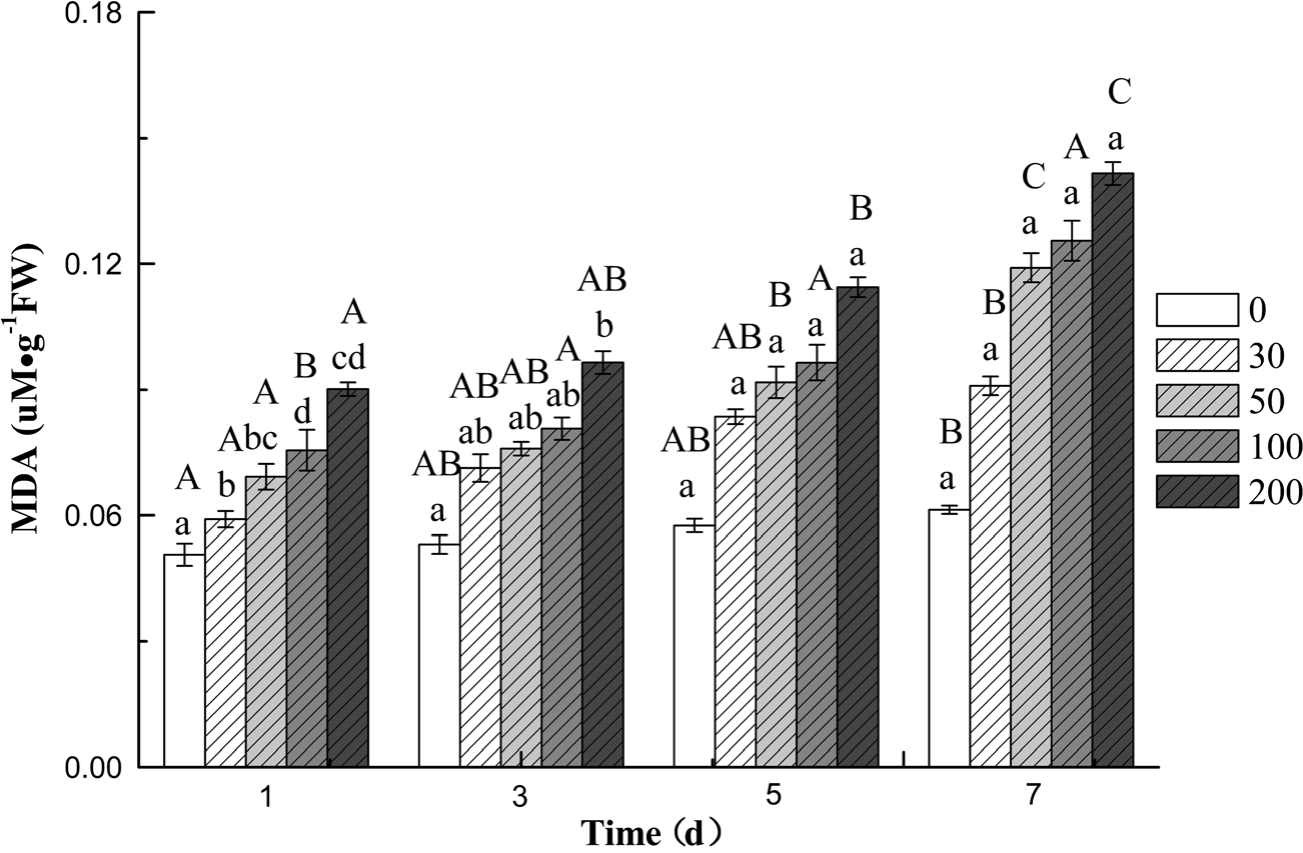
The effect of DBP on the MDA content of *C. sativus* L. cv Jinyan No. 4

**Fig. 5:**
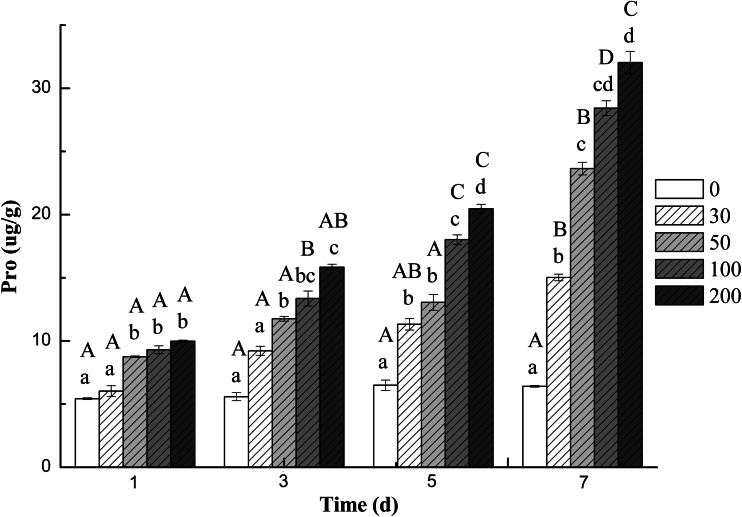
The effect of DBP on the Pro content of *C. sativus* L. cv Jinyan No. 4

The effect of DBP stress on the content of MDA in the roots of cucumber is shown in Fig. 4. The result showed that the content of MDA in roots was enhanced with the extension in time of DBP treatment. The increase was more pronounced with increasing stress by DBP. Compared with the controls, the maximum MDA contents were found at 200 mg L^−1^ DBP on the seventh day. DBP at 200 mg L^−1^ resulted in 2.31 times increase in MDA content in comparison to the control. A similar increasing effect of DBP treatment was also found in Pro content (Fig. 5). The content of Pro was significantly enhanced about 4.99 times compared with the control, under 200 mg L^−1^ DBP treatment.

**Fig. 6:**
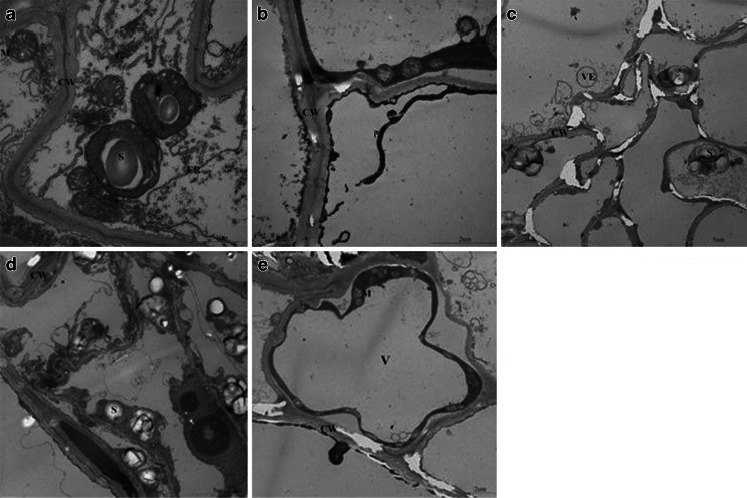
Ultrastructural micrographs of cucumber root showed the typical cellular ultrastructure of root cells with or without DBP

Ultrastructural micrographs of cucumber root showed the typical cellular ultrastructure of root cells with or without DBP (Fig.6, a–e). In well-defined cellular organelles, the cell walls were closely linked. The mitochondria of control plants exhibited an integrated structure. The endoplasmic reticulum and golgi apparatus were clearly visible. Starch grains displayed a spherical shape. Compared with untreated seedlings, the effect of DBP stress on the ultrastructure was much. Root cells became deteriorated, swollen vesicles appeared, endoplasmic reticulum became blurred, and the number of golgi apparatus decreased. Specifically, the mitochondria gradually swelled and inner cristae became invisible under DBP stress. Inner cristae in mitochondria even disintegrated eventually in some cells. Plasmolysis was observed in some cells and the plasma membrane had ruptured. Moreover, compared with control, a great number of starch grains were detected within the cells.

## Discussion

Normally, reactive oxygen species (ROS) levels are in a dynamic balance with the antioxidant enzyme level. However, if the free radicals induced directly or indirectly by xenobiotic pollutants were accumulated in the body, the natural antioxidant defenses could be damaged. The superfluous ROS could result in severe sub-cellular injuries (Nel et al. 2006). The enzymes SOD, CAT, and POD, and non-enzymatic substances such as MDA and Pro were reported to be involved in the detoxification of O_2_^−^· (SOD) and H_2_O_2_ (CAT, POD), and prevented the formation of ROS (Wen et al. 2011).

SOD was the first enzyme in the detoxifying process that converts O_2_^−^· radicals to H_2_O_2_ at a very rapid rate (Hasan et al. 2009). According to the current study, SOD activities presented a first rising then falling trend. It might be due to increase in production of superoxide which leads to increase in genes encoding SOD, under the stress of DBP (Mishra et al. 2006a, b; Zhang et al. 2009). That excessive ROS produced along with the extension of processing time could decline the SOD activities (CHO and SEO 2005). If ROS generation exceeded the elimination ability of antioxidant enzymes, activities of antioxidant enzymes in plants were decreased (Li et al. 2011). The major function of CAT was documented to metabolize the peroxide liberated in the peroxisome following the conversion of glycolate during photorespiration (Qureshi et al. 2007). We noticed that with the extension of exposure time and increasing concentration of DBP, CAT activities of cucumber seedling showed more obvious decrease than that of SOD and POD, which might be due to the accumulation of H_2_O_2_, the product of free radical could restrained the activity of fermentation. Previous observations have proved that PAEs could overproduce H_2_O_2_ (Zhang et al. 2014; Bai et al. 2009). Moreover, the formation of H_2_O_2_ was also detected under the impact of other pollutants (Sanchez-Moreiras and Reigosa 2005; Li et al. 2013; Choudhary et al. 2012).

POD was known to play an important role in oxidative stress conditions. Induction of POD activity was documented under many stress conditions such as high temperature, NaCl stress, and toxic concentrations of Pb, Zn, Cu, and Cd (Radotic et al. 2000a, b). In the present investigation, enhancement in POD activity was observed due to the duration of pollutant stress, which was in agreement with the early findings of Odjegba and Fasidi (2007). The phenomenon could be speculated by the fact that under DBP stress, production of POD increased, and existing enzyme pools were activated or expression of genes encoding POD was increased.

Meanwhile, activities of SOD, CAT, and POD in roots continuously increased within the roots and indicated that the cucumber could resist lower-concentration DBP stress (<50 mg L^−1^) by activating the antioxidant defense system. The findings suggested that the antioxidant defense system in roots eliminated ROS efficiently at low-concentration DBP, and provided protection from oxidative stress. This observation was consistent with that obtained with the leaves of cucumber seedling treated with DEP (Zhang et al. 2014). Qu’s research also got the same conclusion (Qu et al. 2010). In their study, the toxic effects of different concentrations (0.1–200 mg/L) of perfluorooctane sulfonate on wheat were investigated. Their results showed that low concentrations of perfluorooctane sulfonate (0.1–10 mg L^−1^) induced SOD and POD activities in wheat roots and leaves, a high concentration of perfluorooctane sulfonate (200 mg L^−1^) inhibited the activities of SOD and POD.

In this study, CAT activity in roots decreased at the high concentrations of DBP along with time. It indicated that ROS elimination of CAT was limited. DBP could induce higher SOD and POD activities than CAT activity. The result suggested that SOD and POD exhibited a better defense mechanism against DBP-induced oxidative damage in the roots of cucumber seeding.

The imbalance between ROS generation and removal results in damage to DNA, lipids, and proteins (Valko et al. 2006). Lipid peroxidation of polyunsaturated fatty acids is initiated due to the formation of ROS resulting in reactive carbonyl compounds production. MDA is among secondary products of lipid peroxidation and is extensively studied as a potential biomarker for oxidative stress (Nielsen et al. 1997). MDA concentration has been measured to examine oxidative stress and as an indicator of oxidative imbalance during the onset of many diseases (Niki 2008). As depicted in Fig. 2 the MDA content changed slightly during exposure. With increased concentration of DBP and exposure duration, the MDA content increased significantly. These results also indicated that DBP resulted in an increase of ROS. ROS, in turn, stimulated the response of antioxidant defenses and resulted in a decreased in SOD, CAT, and POD activities. Demiral and Türkan (2005) reported that slight decline of SOD and CAT activity was found in roots of IR-28 under increased salt concentrations, respectively. The changes in the MDA contents were approximately same as those observed by Bai et al. (2009) and Liu et al. (2012b).

Pro was generally assumed to serve as a physiologically compatible solute that increases as needed to maintain a favorable osmotic potential between the cell and its surroundings (Pollard and Wyn Jones 1979). The Pro contents in roots had the same trend to MDA. In salt-tolerant and relatively salt-tolerant plants like *C. sativus* L. cv Jinyan No. 4 (Koca et al. 2007), *Beta vulgaris* (Gzik 1996) and *Brassica juncea* (Jain et al. 1991), sharp increases in proline levels were reported under the effect of salinity. The accumulation of this amino acid may be part of a general adaptation to adverse environmental conditions (Delauney and Verma 1993).

The roots ultrastructural study showed that the organelles were obviously affected. In the present study, we observed some organelles in cucumber roots showed apparent changes under the impact of DBP. Damage to plasma membrane and plasmolysis phenomenon might be because of the increased content of MDA and Pro. The increase of MDA content at all treatment levels might result in cellular injury which causes organelle damage (Forman et al. 2002; Lara-Nunez et al. 2006). Pro is thought to play a cardinal role as an osmoregulatory solute in plants subjected to hyperosmotic stress, primarily drought and soil salinity (Delauney and Verma 1993). The content of MDA and Pro was increased in this study which indicated that changes of MDA and Pro were the important reasons leading to the damage of plasma membrane and plasmolysis.

Mitochondrion is one of the important sources of H_2_O_2_ in plant cells (Hernandez et al. 2010; Rhoads et al. 2006). According to Yamane et al. (2012), H_2_O_2_ generated from the electron transfer chains damaged mitochondrial cristae under the influence of salinity. Similar findings were observed in root tip cells of soybean under Al stress (Yu et al. 2011). It was founds that the mitochondria in root tip cells were irregular in shape and swollen; the cristae system was a little bit distorted under Al stress. This variation was caused by the decline of CAT and POD activities. Plant cell vacuoles are one of the most variable multifunctional organelles. The vacuoles of root cells have storage functions (Zaalishvili et al. 2002). Enlargement of the vacuoles could be observed in this present work. The results obtained are in agreement with previous reports of Dauda et al. (2009), when exposing transgenic cotton to Ca. Increase in the size of vacuoles might be helpful in preventing the circulation of DBP in the cytosol and forcing them into a limited area (Sanita and Gabbrielli 1999). Numerous starch grains were discovered in root cells. The concentration of these grains was possibly due to the root cells not adapting to DBP and therefore presenting the aging change. Santos et al. (2013) and Bouzon et al. (2012) also found this appearance when mesophyll cells were exposed to Cd.

In conclusion, *C. sativus* L. cv Jinyan No. 4 was found to be sensitive to DBP. DBP stress significantly affected enzyme activities, lipid peroxidation, and caused damage to root cells. Cucumber could defend lower-concentration DBP stress (<50 mg L^−1^) effectively by activating the antioxidant defense system. Compared to CAT, SOD, and POD exhibited a better defense mechanism against oxidative damage induced by DBP. This study would be helpful to understand the toxicity effect of PAEs to plant.

## Abbreviations

CAT: catalase
DBP: di-*n*-butyl phthalate
PAEs: phthalic acid esters
PBS: phosphate-buffered saline solution
POD: peroxidase
Pro: proline
MDA: malondialdehyde
ROS: reactive oxygen species
SOD: superoxide dismutase
